# A nutritional supplement taken during preconception and pregnancy influences human milk macronutrients in women with overweight/obesity and gestational diabetes mellitus

**DOI:** 10.3389/fnut.2023.1282376

**Published:** 2023-10-17

**Authors:** Soo Min Han, José G. B. Derraik, Mark H. Vickers, Surabhi Devaraj, Fang Huang, Wei Wei Pang, Keith M. Godfrey, Shiao-Yng Chan, Sagar K. Thakkar, Wayne S. Cutfield, NiPPeR Study Group, Benjamin B. Albert, Shelia J. Barton, Aristea Binia, Mary Cavanagh, Hsin Fang Chang, Yap Seng Chong, Mary F. Chong, Cathryn Conlon, Cyrus Cooper, Paula Costello, Vanessa Cox, Christine Creagh, Marysia Depczynski, Sarah El-Heis, Judith Hammond, Zhang Han, Nicholas C. Harvey, Mrunalini Jagtap, Timothy Kenealy, Heidi Nield, Justin M. O’Sullivan, Gernalia Satianegara, Irma Silva-Zolezzi, Shu E. Soh, Vicky Tay, Rachael Taylor, Elizabeth Tham, Philip Titcombe, Clare Wall, Ray Wong, Gladys Woon

**Affiliations:** ^1^Liggins Institute, The University of Auckland, Auckland, New Zealand; ^2^Department of Paediatrics: Child and Youth Health, Faculty of Medical and Health Sciences, The University of Auckland, Auckland, New Zealand; ^3^Nestlé Research, Société des Produits Nestlé S.A., Singapore, Singapore; ^4^Nestlé Research, Société des Produits Nestlé S.A., Beijing, China; ^5^Global Centre for Asian Women’s Health, Dean’s Office, Yong Loo Lin School of Medicine, National University of Singapore, Singapore, Singapore; ^6^Department of Obstetrics and Gynaecology, Yong Loo Lin School of Medicine, National University of Singapore and National University Health System, Singapore, Singapore; ^7^MRC Lifecourse Epidemiology Centre, University of Southampton, Southampton, United Kingdom; ^8^NIHR Southampton Biomedical Research Centre, University Hospital Southampton NHS Foundation Trust and University of Southampton, Southampton, United Kingdom; ^9^Singapore Institute for Clinical Sciences, Agency for Science, Technology and Research, Singapore, Singapore; ^10^A Better Start—National Science Challenge, The University of Auckland, Auckland, New Zealand

**Keywords:** human milk, macronutrients, gestational diabetes mellitus, maternal BMI, maternal nutrition

## Abstract

**Rational:**

Maternal overweight/obesity and gestational diabetes mellitus (GDM) are associated with an increased risk of their offspring developing overweight/obesity or type 2 diabetes later in life. However, the impacts of maternal overweight/obesity and dysglycemia on human milk (HM) macronutrient composition are not well understood.

**Objective:**

Through a double-blind randomised controlled trial, we investigated the effects of maternal supplementation from preconception throughout pregnancy until birth on HM macronutrient concentrations, in association with maternal and infant factors including maternal pre-pregnancy body mass index (BMI) and GDM status. In addition, we aimed to characterise longitudinal changes in HM macronutrients.

**Methods:**

The control supplement contained calcium, iodine, iron, β-carotene, and folic acid. The intervention supplement additionally contained zinc, vitamins B_2_, B_6_, B_12_, and D_3_, probiotics, and myo-inositol. HM samples were collected across seven time points from 1 week to 12 months from Singapore and/or New Zealand. HM macronutrient concentrations were measured using a MIRIS Human Milk Analyser. Potential differences in HM macronutrient concentrations were assessed using linear mixed models with a repeated measures design.

**Results:**

Overall, HM macronutrient concentrations were similar between control and intervention groups. Among the control group, overweight/obesity and GDM were associated with higher HM fat and energy concentrations over the first 3 months. Such associations were not observed among the intervention group. Of note, mothers with GDM in the intervention group had lower HM fat by 10% (*p* = 0.049) and energy by 6% (*p* = 0.029) than mothers with GDM in the control group. Longitudinal changes in HM macronutrient concentrations over 12 months of lactation in New Zealand showed that HM fat and energy decreased in the first 6 months then increased until 12 months. HM lactose gradually decreased from 1 week to 12 months while crude protein decreased from 1 week to 6 months then remained relatively constant until 12 months of lactation.

**Conclusion:**

Maternal overweight/obesity or GDM were associated with increased HM fat and energy levels. We speculate the intervention taken during preconception and pregnancy altered the impact of maternal BMI or GDM status on HM macronutrient composition. Further studies are required to identify the mechanisms underlying altered HM macronutrient concentration in the intervention group and to determine any long-term effects on offspring health.

**Clinical trial registration:**

ClinicalTrials.gov, NCT02509988, Universal Trial Number U1111-1171-8056. Registered on 16 July 2015. This is an academic-led study by the EpiGen Global Research Consortium.

## 1. Introduction

Human milk (HM) provides the essential nutrients and bioactive factors infants need for growth and development (1, 2). The World Health Organisation (WHO) recommends infants to be exclusively breastfed for at least 6 months (3), which has been associated with long-term infant outcomes including lower risks of obesity (4, 5) type 2 diabetes (6), infections (7), allergies, and asthma (8, 9), and improved neurodevelopmental outcomes (10). Some HM components have been associated with specific outcomes. For example, HM oligosaccharides have been associated with altered risks of allergies or infections (11, 12) and cognitive developmental scores in infants (13). Further, a recent pre-clinical study demonstrated that myo-inositol promotes neuronal connectivity, providing insights into the role of myo-inositol in infant brain development (14). HM macronutrient composition has been associated with infant body composition up to 12 months of age (15, 16). However, there is limited and inconsistent evidence in this area.

In addition to bioactive compounds such as immunological components and growth and metabolic hormones, HM provides nutrients and energy for infant growth and development. HM contains approximately 3.8% fat, 7% lactose, and 1% protein, each contributing about 50%, 40–45%, and 5–6% to total energy, respectively (17, 18). Infant formula contains proportions of macronutrients similar to HM, with fat providing approximately 45–50% of total energy, carbohydrate 40–45%, and protein 8–12% (18). While infant formula is a standardised solution over specific age ranges, HM is a dynamic compound, changing during a feed (19), throughout the day (20), and over the course of lactation (19, 21). Moreover, HM macronutrient composition may vary according to a range of maternal and infant factors, but these associations are not well understood. With the exception of fatty acids, maternal diet is reported to have no association with HM macronutrients (22). Positive associations have been reported between maternal body mass index (BMI) and HM fat and energy (23–26), and between maternal age and HM fat and carbohydrate (27–30). In addition, negative associations have been reported between infant gestational age and HM protein (27, 31), fat, and lactose (21, 30, 32). There have been inconsistent observations on the influence of maternal GDM status (27, 33, 34), infant sex (26, 35–38), parity (28, 39), and mode of delivery (24, 32, 36, 40) on HM macronutrient composition.

The Nutritional Intervention Preconception and During Pregnancy to Maintain Healthy Glucose Metabolism and Offspring Health (NiPPeR) study was a double-blind, randomised controlled trial investigating the effects of a nutritional supplement during preconception and pregnancy on maternal pregnancy outcomes and infant growth (41). Adequate maternal micronutrient status during pregnancy and lactation is essential for both mothers and infants (42). Micronutrient supplementation during pregnancy has been associated with lower risks of adverse pregnancy outcomes (43). For example, folic acid supplementation decreased occurrence of neural tube defect in the offspring (44); a combined supplementation of folic acid and iron reduced the risk of post-partum haemorrhage (45); calcium supplementation lowered the risk of pre-eclampsia (46); and vitamin D supplementation was associated with reduced risks of pre-eclampsia (47). In our recent publications, we showed that micronutrient supplementation during pre-conception and pregnancy increased HM concentrations of zinc (48) and vitamin D (49) in the first 3 months of lactation. However, there remains a paucity of data on the potential impacts of micronutrient supplementation during preconception and pregnancy on HM macronutrient composition during lactation. Therefore, the aim of this study was to describe HM macronutrient composition following nutritional supplementation during preconception and pregnancy in association with maternal and infant factors including maternal pre-pregnancy BMI and GDM status.

## 2. Materials and methods

### 2.1. Study design

A detailed protocol for the NiPPeR study (ClinicalTrials.gov, identifier: NCT02509988, Universal Trial Number U1111-1171-8056; registered on July 16, 2015) has been published previously (41). Briefly, the NiPPeR study was a double-blind, randomised, controlled trial investigating the effects of a nutritional supplement taken from preconception and during pregnancy on maternal pregnancy and infant outcomes. The control supplement comprised of micronutrients that are present in commonly used pregnancy supplements: calcium, iron, iodine, folic acid, and vitamin A. The NiPPeR intervention supplement additionally contained zinc, vitamin B_2_, vitamin B_6_, vitamin B_12_, vitamin D_3_, myo-inositol, and probiotics (Table 1). The study supplements were packaged as a powder form in sachets and were taken twice daily, as a drink reconstituted with water. Adherence to the study protocol was assessed by sachet counting, with good adherence defined as at least 60% of the sachets consumed (53). The primary outcome of gestational glycaemia and the secondary outcome of GDM did not differ between control and intervention groups (53). The study was conducted in Southampton (United Kingdom), Singapore, and Auckland (New Zealand) and ethics approval was obtained at each site [Southampton—Health Research Authority National Research Ethics Service Committee South Central Research Ethics Committee (15/SC/0142); Singapore—the National Healthcare Group Domain Specific Review Board (2015/00205); and New Zealand—Northern A Health and Disability Ethics Committee (15/NTA/21)]. All participants provided written informed consent.

**Table 1:** Detailed nutrient composition of the intervention and control drinks in the NiPPeR study.

Group	Nutrient	Intervention	Control	Daily dose	Recommended range^#^
Minerals	Calcium	✓	✓	150 mg	700–1,300 mg
	Iodine	✓	✓	150 μg	140–220 μg
	Iron	✓	✓	12 mg	14.8–27.0 mg
	Zinc	✓	✘	10 mg	7–15 mg
Vitamins	A (β-carotene)	✓	✓	720 μg	700–750 μg
	B_2_ (Riboflavin)	✓	✘	1.8 mg	1.38–1.46 mg
	B_6_ (Pyridoxine)	✓	✘	2.6 mg	1.2–1.9 mg
	B_9_ (Folic acid)	✓	✓	400 μg	300–600 μg
	B_12_ (Cobalamin)	✓	✘	5.2 μg	1.5–2.6 μg
	D_3_ (Cholecalciferol)	✓	✘	400 IU (10 μg)	5–10 μg
Other	Myo-inositol	✓	✘	4 g	n/a
	Lactobacillus rhamnosus*	✓	✘	>1 × 10^9^ CFU	n/a
	*Bifidobacterium animalis* ssp. *lactis*^†^	✓	✘	>1 × 10^9^ CFU	n/a

^#^Recommended ranges for daily intake during pregnancy according to the reference nutrient intake for the United Kingdom (50), recommended dietary allowance for Singapore (51), and recommended daily intake for New Zealand (52). ^*^NCC 4007 (CGMCC 1.3724). ^†^NCC 2818 (CNCM I-3446). CFU, Colony-forming units; n/a, Not applicable. Table reproduced from Han et al. (48).

### 2.2. Study participants

Participants were recruited by self-referral after study information was distributed through local and social media advertisements. The key inclusion criteria were women aged 18–38 years who were planning to conceive within 6 months. The full inclusion, exclusion, and withdrawal criteria have been reported previously (41) and are provided in Supplementary Table 1. Eligible participants were randomised in a 1:1 ratio to either the control or the intervention group through the electronic study database (41), and stratified by site and ethnicity to ensure balanced allocation of participants.

### 2.3. Human milk sample collection

**Figure 1:**
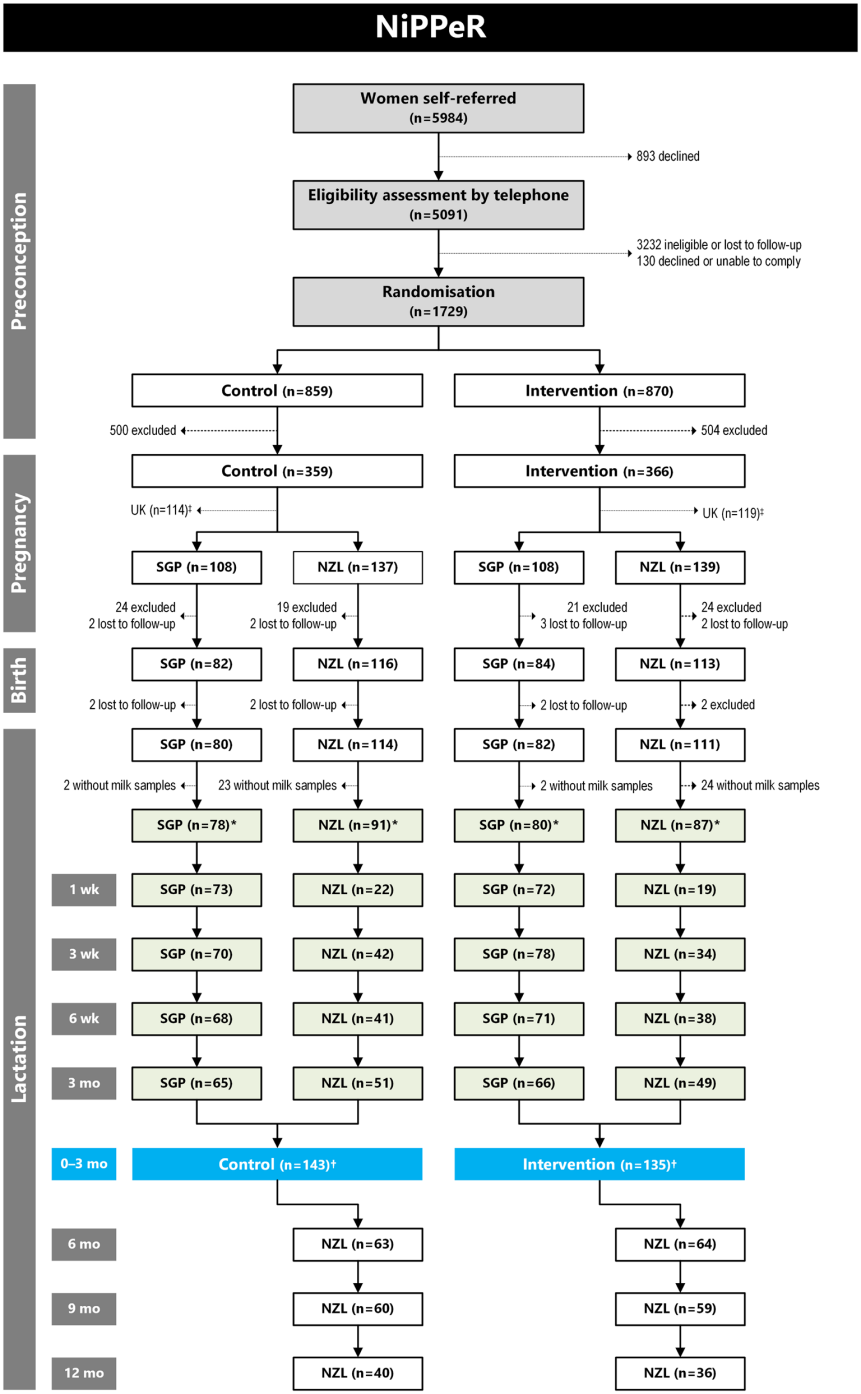
CONSORT diagram for the number of human milk (HM) samples analysed for macronutrients in the NiPPeR study. Reasons for exclusion during the preconception phase have been published previously (53), while reasons for exclusion during pregnancy and birth in Singapore (SGP) and New Zealand (NZL) are provided in Supplementary Table 2. ^‡^There were no HM samples collected in the United Kingdom (UK), so all participants from that site were excluded from this diagram. ^*^Number of participants who provided at least one HM sample during the first 12 months of lactation. ^†^Number of participants who provided at least one HM sample during the first 3 months of lactation. Diagram adapted from Han et al. (48).

HM samples were collected in Singapore until 3 months (July 2016 to March 2019) and in New Zealand until 12 months of lactation (May 2017 to November 2019); practical constraints precluded collection in the United Kingdom (Figure 1). Samples were collected at four time points common to both sites: 1 week (±3 days), 3 weeks (±5 days), 6 weeks (±5 days), and 3 months (±10 days). In New Zealand, there were additional HM collections at 6 months (±14 days), 9 months (±14 days), and 12 months (±14 days); seven time points in total. In Singapore, samples were only collected until 3 months due to logistical constraints. Mothers were asked to refrain from breastfeeding for 2 hours prior to sample collection from the breast where samples would be collected. Under the supervision of trained staff, whole HM samples were collected in the morning from a single breast pumped for 15 minutes using an Ameda Lactaline breast pump (Ameda, Inc., Murarrie, Australia) or until fully emptied. Following collection, HM samples were homogenised, then stored at −80°C until analysis. HM samples were not collected if the mother had ceased breastfeeding, her milk supply was low, or there were complications with milk expression. Figure 1 shows the number of samples analysed at each time point.

### 2.4. Human milk macronutrient analyses

HM fat, energy, lactose, and crude protein were quantified via infrared transmission spectroscopy using a Miris Human Milk Analyser (HMA; Miris, Uppsala, Sweden) following the manufacturer’s protocol. 1–3 mL of frozen HM samples were slowly thawed overnight at 4°C. Prior to analysis, they were warmed to 40°C in a water bath and homogenised using a Miris Ultrasonic Processor (Miris, Uppsala, Sweden) at a processing time of 1.5 s/mL. Quality control was performed using a HM sample with known macronutrient composition after every 20 samples and clean and check performances were done every 10 samples.

### 2.5. Statistical analyses

Descriptive statistics were calculated for maternal, infant, and birth-related characteristics. For intergroup comparisons, the independent samples *t-*test was used for continuous variables, and Chi-square tests were used to compare categorical variables between randomisation groups and between sites. For HM macronutrient measurements, samples with one or more macronutrient measurement value of ‘0’ were excluded from analysis due to a possible risk of sample dilution. In addition, we adopted a conservative approach to removing outliers from analyses, excluding values outside the range of the mean ± 5 × standard deviations (SD; Supplementary Table 3).

Potential intervention effects on HM macronutrient concentrations were examined on the samples collected in the first 3 months of lactation only, collected in both Singapore and New Zealand. Key parameters included in linear mixed models were randomisation group, visit, their interaction term (group*visit), study site, maternal pre-pregnancy BMI, infant gestational age at birth, and adherence to the study supplements as a continuous variable. Participant study ID number was also included as a random factor to account for the repeated measurements. Subgroup analyses were performed to examine potential intervention effects over the first 3 months of lactation separately for Singapore and New Zealand. Temporal changes in HM macronutrients from 1 week to 12 months of lactation are described for the New Zealand site only as samples from the later time points were not available in Singapore.

As secondary analyses, potential interactions between the intervention and maternal metabolic status (pre-pregnancy BMI or GDM) and their associations with HM macronutrients were examined for the first 3 months of lactation in fully adjusted models. Pre-pregnancy BMI was defined using the WHO classification (54): underweight <18.5 kg/m^2^, normal weight 18.5–24.9 kg/m^2^, overweight 25.0–29.9 kg/m^2^, and obesity ≥30 kg/m^2^. Maternal GDM status was determined by an oral glucose tolerance test at 28 weeks gestation as defined by the International Association of Diabetes and Pregnancy Study Groups (IADPSG) criteria (55).

As exploratory analyses, potential associations between other maternal and infant factors with HM macronutrients were assessed. The mean HM macronutrient concentrations in the first 3 months were compared between binary categories of maternal ethnicity (Non-Asian vs. Asian), maternal age (< 35 vs. ≥ 35 years old), delivery mode (Vaginal vs. Caesarean-section), parity (Primiparous vs. Multiparous), infant sex (Male vs. Female), and infant gestational age (Term/Post-term ≥37 weeks vs. Preterm <37 weeks). Interactions between each of these maternal/infant factors and the intervention group were tested but none were statistically significant.

Study outcomes are reported as the back-transformed least-squares means (i.e., adjusted means) for each group or the adjusted mean difference (aMD) between groups and their respective 95% confidence intervals (CI). The aMD for back-transformed values represent proportional differences between the comparison groups. Statistical analyses was carried out using IBM SPSS Statistics for Windows, Version 26 (IBM Corp., Armonk, NY, United States) and SAS Version 9.4 (SAS Institute Inc., Cary, NC, United States). Graphs were created with GraphPad Prism version 9.3.1 (GraphPad Software, San Diego, California, United States). All statistical tests were two-sided with significance maintained at *p* < 0.05, without adjustments for multiple comparisons or imputation of missing values.

## 3. Results

### 3.1. Study population

In total, 336 of 387 participants (86.8%) from Singapore and New Zealand sites who continued to the postpartum stage of the study provided at least one HM sample in the first 12 months of lactation (Figure 1). Maternal pre-pregnancy BMI (*p* = 0.047) and passive smoking rates (*p* = 0.035) were lower in the intervention group compared with the control. Other baseline and perinatal characteristics, including GDM rates, were similar between control and intervention groups (Table 2). In Singapore, most participants were of Chinese ethnicity (78.5%), while in New Zealand, most were White Caucasian (75.8%; *p* < 0.001). Compared to the New Zealand cohort, the Singapore participants had lower pre-pregnancy BMI (*p* < 0.001), a higher GDM rate (*p* < 0.001), more vaginal deliveries (*p* = 0.023), lower infant birth weight (*p* < 0.001), and shorter gestation (*p* < 0.001), but the incidence of preterm delivery was not different between the two sites (Supplementary Table 4).

**Table 2:** Baseline and perinatal characteristics of participants in the control and intervention groups in the NiPPeR study who provided at least one human milk sample during 12 months of lactation.

	Overall (*n* = 336)		*p*-value
	Control	Intervention	
*n*	169 (50.3%)	167 (49.7%)	
Adherence (%)	89.2 (82.9–95.9)	90.4 (82.9–96.0)	n.s.
Duration of supplementation (days)	405.2 ± 105.0	393.4 ± 98.2	n.s.
Ethnicity			
Caucasian	69 (40.8%)	66 (39.5%)	n.s
Chinese	70 (41.4%)	69 (41.3%)	
South Asian	10 (5.9%)	10 (6.0%)	
Malay	10 (5.9%)	10 (6.0%)	
Other	10 (5.9%)	12 (7.2%)	
Maternal age			
Age at delivery (years)	31.9 ± 2.9	32.3 ± 3.2	n.s.
<35	145 (85.8%)	130 (77.8%)	n.s
≥35	24 (14.2%)	37 (22.2%)	
Maternal pre-pregnancy BMI status			
BMI (kg/m^2^)	24.4 ± 5.2	23.3 ± 4.4	0.047
Underweight/ normal weight	115 (68.0%)	128 (76.6%)	n.s.
Overweight	31 (18.3%)	25 (15.0%)	
Obesity	23 (13.6%)	13 (7.8%)	
Missing	–	1 (0.6%)	
Highest level of education			
Bachelor’s degree or higher	136 (80.5%)	135 (80.8%)	n.s.
Lesser qualification^*^	33 (19.5%)	32 (19.2%)	
Household income quintile			
5 (lowest)	4 (2.4%)	1 (0.6%)	n.s.
4	12 (7.1%)	16 (9.6%)	
3	43 (25.4%)	43 (25.7%)	
2	60 (35.5%)	55 (32.9%)	
1 (highest)	44 (26.0%)	42 (25.1%)	
Missing	6 (3.6%)	10 (6.0%)	
Smoking during pregnancy			
None	133 (78.7%)	148 (88.6%)	0.035
Passive	33 (19.5%)	16 (9.6%)	
Active	3 (1.8%)	3 (1.8%)	
GDM			
No GDM	123 (72.8%)	119 (71.3%)	n.s.
GDM	41 (23.4%)	42 (25.1%)	
Excluded	5 (3.0%)	6 (3.6%)	
Mode of delivery			
Vaginal delivery	124 (73.4%)	119 (71.3%)	n.s.
Caesarean section	45 (26.6%)	48 (28.7%)	
Infant birth weight			
Birth weight (kg)	3.24 ± 0.54	3.23 ± 0.53	n.s.
Appropriate for gestational age	143 (84.6%)	142 (85.0%)	n.s.
Large for gestational age	10 (5.9%)	6 (3.6%)	
Small for gestational age	16 (9.5%)	19 (11.4%)	
Infant gestational age			
Gestational age (weeks)	39.2 ± 1.6	39.2 ± 1.5	n.s.
Preterm	14 (8.3%)	11 (6.6%)	n.s.
Term or post-term	155 (91.7%)	156 (93.4%)	
Parity			
Primiparous	113 (66.9%)	95 (56.9%)	n.s
Multiparous	56 (33.1%)	72 (43.1%)	
Infant sex			
Male	75 (44.4%)	79 (47.3%)	n.s.
Female	94 (55.6%)	88 (52.7%)	

Data are *n* (%), mean ± SD, or median (Q1–Q3). *p*-values from independent samples *t*-test for continuous variables or Chi-square tests for categorical variables. Adherence to the study protocol was determined by sachet counting. Duration of supplementation calculated by counting the number of days from randomisation date to delivery date. Body mass index (BMI) status was defined as per World Health Organisation: Underweight /normal weight < 25.0 kg/m^2^, overweight 25.0–29.99 kg/m^2^, obesity ≥ 30.0 kg/m^2^ (54). Gestational diabetes mellitus (GDM) was defined by International Association of Diabetes and Pregnancy Study Groups (IADPSG) criteria (55). Birth weight categories were determined using the Royal College of Paediatrics and Child Health 2009 U.K.-World Health Organisation growth charts (56). Gestational age was determined using a pre-specified algorithm as previously described (57) with preterm defined as birth < 37 weeks of gestation, and term or post-term as birth at ≥ 37 weeks of gestation. ^*^Including incomplete and complete high school qualifications, and other tertiary level qualifications below bachelors (e.g., diploma or certificate). n.s., not statistically significant at *p*<0.05.

### 3.2. Impact of intervention on HM macronutrients

**Figure 2:**
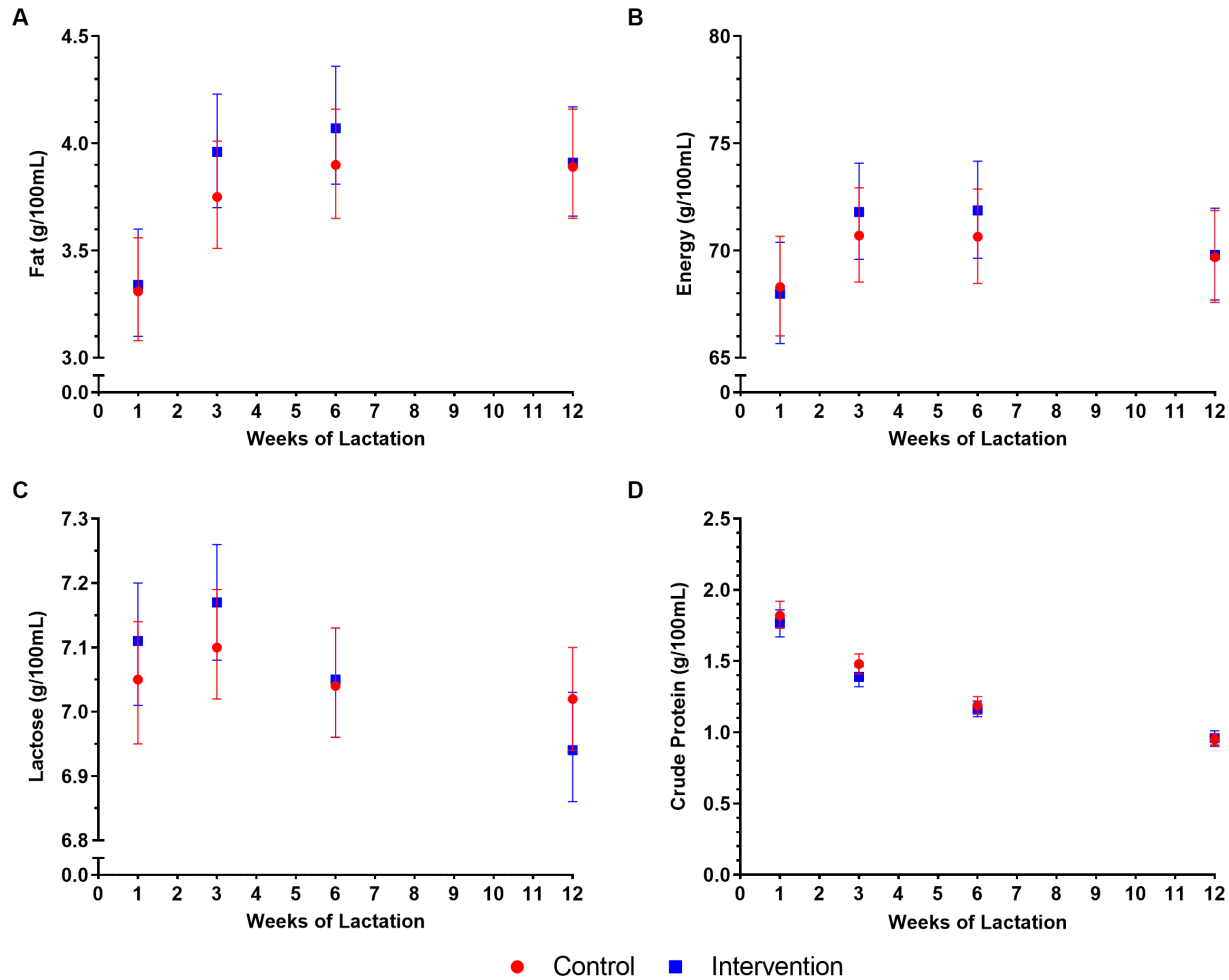
Macronutrient concentrations in human milk (HM) in control and intervention groups in the NiPPeR study, during the first 3 months of lactation: **(A)** fat, **(B)** energy, **(C)** lactose, and **(D)** crude protein. Data are the least-squares means (i.e., adjusted means) for each group, adjusted for randomisation group, visit, an interaction term (group*visit), study site, maternal pre-pregnancy body mass index, gestational age at birth, and adherence. Error bars represent the respective 95% confidence intervals.

During the first 3 months of lactation, the mean HM macronutrient concentrations did not differ between the intervention and control groups with Singapore and New Zealand sites combined (Figure 2) or when each site was analysed separately (Supplementary Table 5).

### 3.3. Maternal pre-pregnancy BMI and GDM status on HM macronutrients

**Figure 3:**
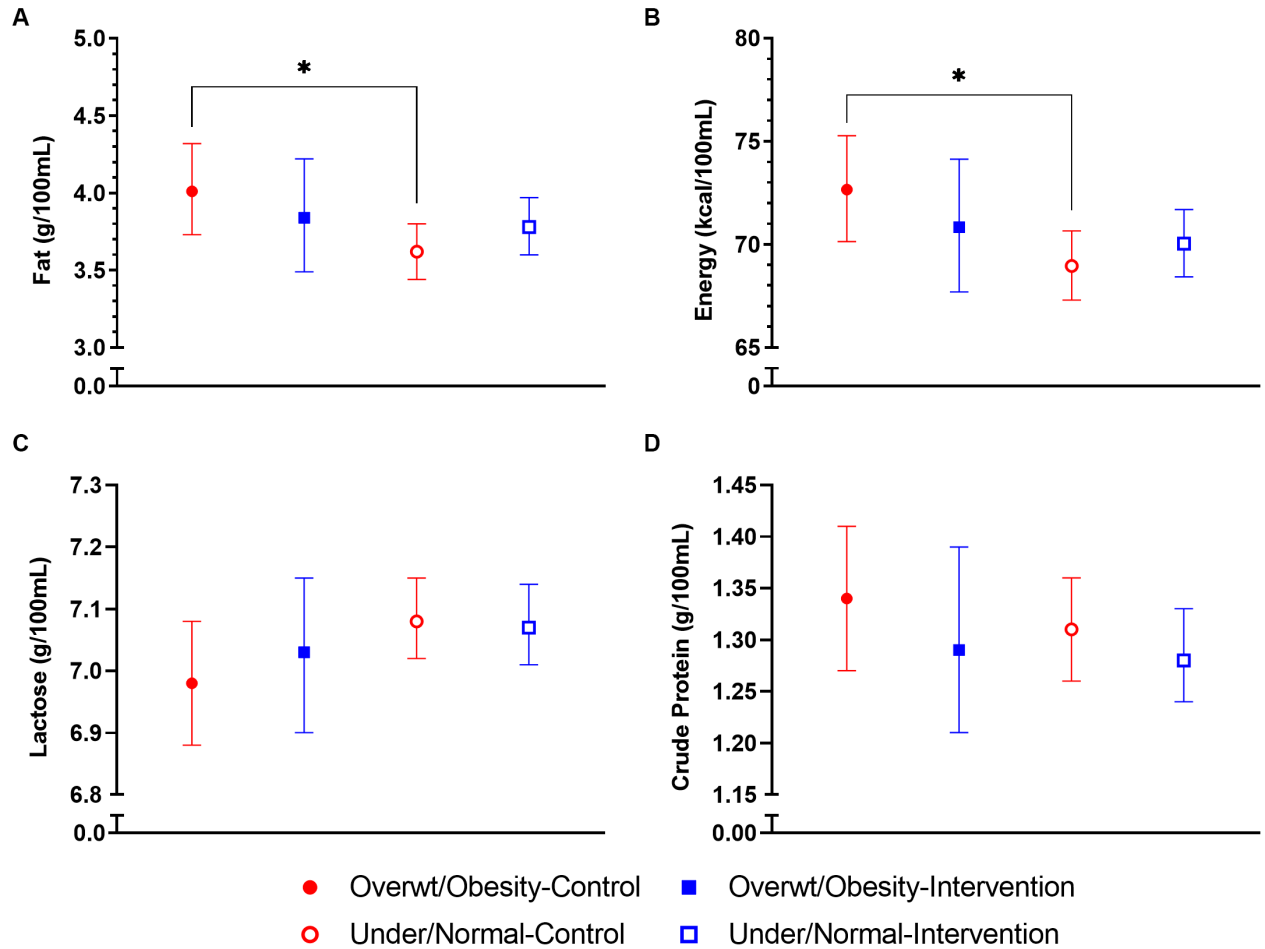
Average macronutrient concentrations in human milk (HM) in control and intervention groups by maternal pre-pregnancy body mass index (BMI) status in the NiPPeR study over the first 3  months of lactation: **(A)** fat, **(B)** energy, **(C)** lactose, and **(D)** crude protein. Data are the least-squares means (i.e., adjusted means) for each group, adjusted for randomisation group, BMI status, group*BMI interaction term, visit, study site, gestational age at birth, and adherence. Error bars represent the respective 95% confidence intervals. ^*^p < 0.05. Overwt/Obesity, Overweight/Obesity BMI; Under/Normal, Underweight/Normal weight BMI.

**Figure 4:**
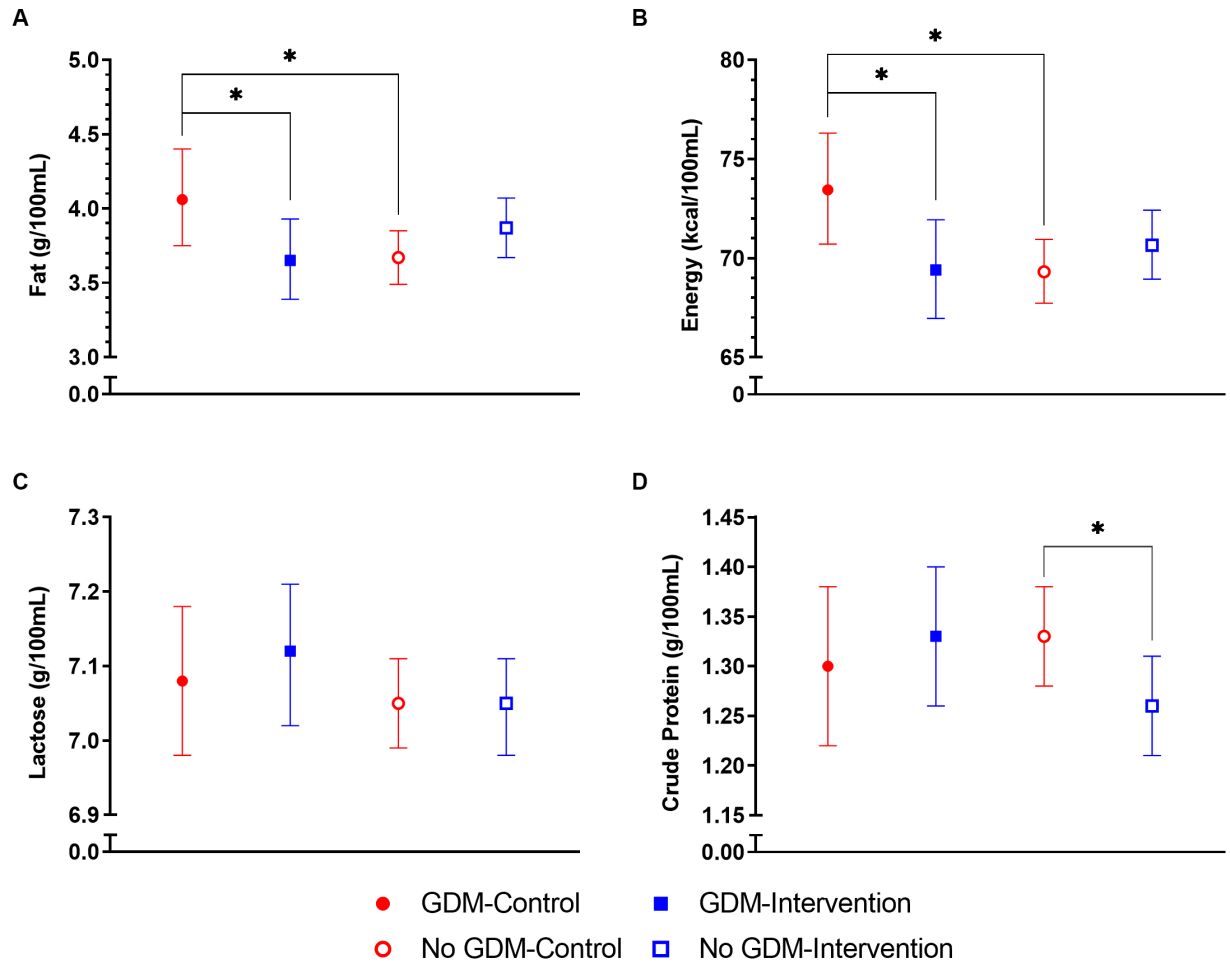
Average macronutrient concentrations in human milk (HM) in control and intervention groups by maternal gestational diabetes mellitus (GDM) status in the NiPPeR study over the first 3 months of lactation: **(A)** fat, **(B)** energy, **(C)** lactose, and **(D)** crude protein. Data are the least-squares means (i.e., adjusted means) for each group, adjusted for randomisation group, GDM status, group*GDM interaction term, visit, study site, gestational age at birth, and adherence. Error bars represent the respective 95% confidence intervals. ^*^p < 0.05.

Among the control group, HM from mothers with overweight/obesity BMI had fat concentrations 11% higher [aMD (95% CI) = 1.11 (1.01, 1.21), *p* = 0.023] and energy content 5% higher [aMD (95% CI) = 1.05 (1.01, 1.10), *p* = 0.017] than mothers with underweight/normal weight BMI over the first 3 months of lactation (Figure 3). Such differences were not observed in the intervention group. Similarly, among the control group, HM fat concentrations were 11% higher [aMD (95% CI) = 1.11 (1.01, 1.22), *p* = 0.030] and energy content 6% higher [aMD (95% CI) = 1.06 (1.01, 1.11), *p* = 0.011] in mothers with GDM compared to those without GDM (Figure 4). Of note, mothers with GDM in the intervention group had HM fat concentrations 10% lower [aMD (95% CI) = 0.90 (0.81, 1.00), *p* = 0.049] and energy content 6% lower [aMD (95% CI) = 0.94 (0.90, 0.99), *p* = 0.029] than mothers with GDM in the control group.

### 3.4. Changes in HM macronutrients over time in New Zealand (0–12 months)

**Figure 5:**
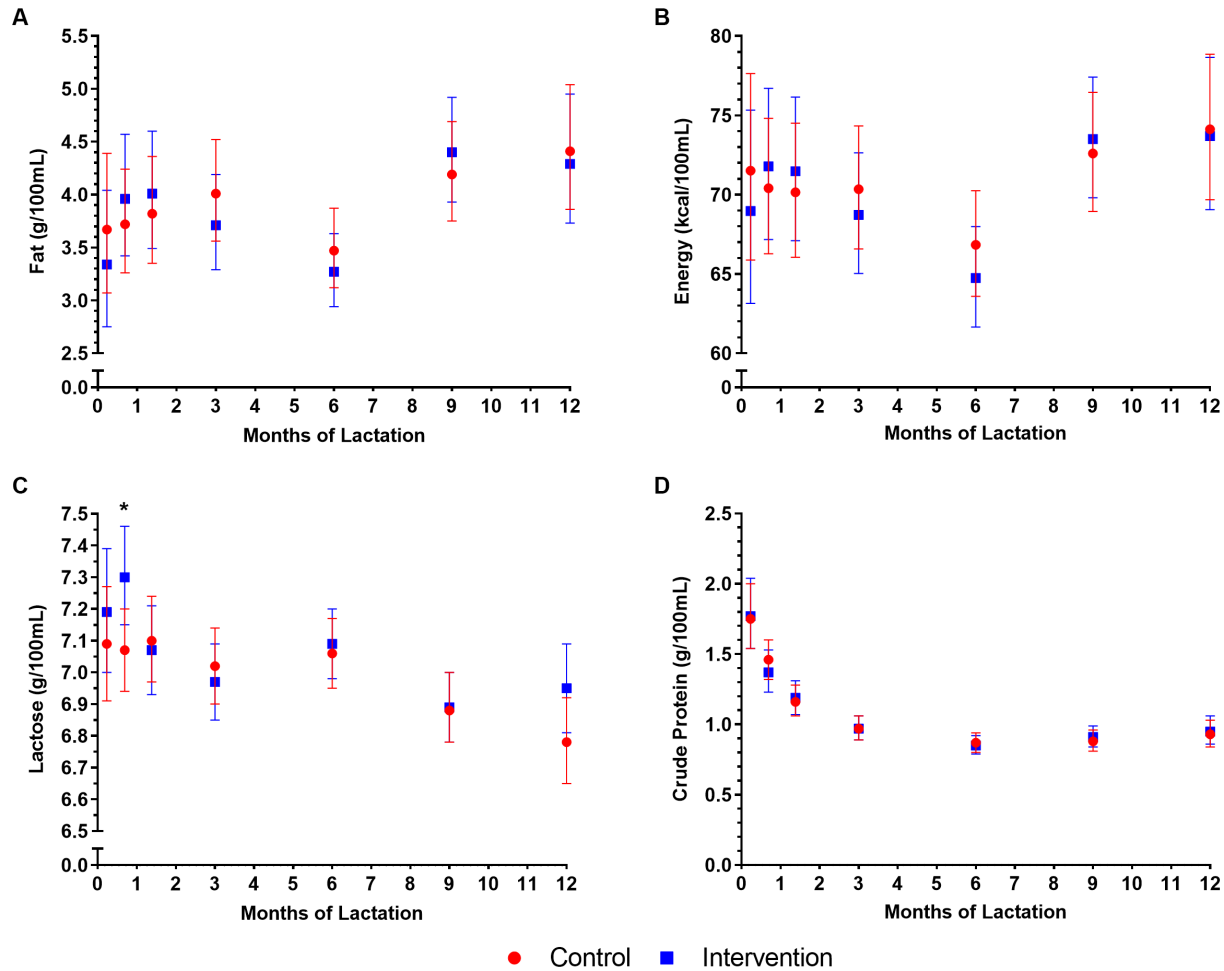
Macronutrient concentrations in human milk (HM) in control and intervention groups in New Zealand in the NiPPeR study during 12 months of lactation: **(A)** fat, **(B)** energy, **(C)** lactose, and **(D)** crude protein. Data are the least-squares means (i.e., adjusted means) for each group, adjusted for randomisation group, visit, an interaction term (group*visit), maternal pre-pregnancy body mass index, gestational age at birth, and adherence. Error bars represent the respective 95% confidence intervals. ^*^p < 0.05 for a difference between intervention and control groups at a given time point.

Among the New Zealand cohort, longitudinal changes in HM macronutrient concentrations were observed in the first 12 months of lactation (Figure 5; Supplementary Table 6). HM fat and energy followed a similar decreasing pattern in the first 6 months, after which they increased until 12 months of lactation (Figures 5A,B). HM lactose gradually decreased from 1 week to 12 months of lactation but there was little intra-individual variation in absolute concentrations during this time (Figure 5C). HM crude protein continuously decreased from 1 week to 6 months, after which it remained relatively constant until 12 months of lactation (Figure 5D).

### 3.5. Other maternal and infant factors and HM macronutrients

HM macronutrient concentrations were associated with some maternal factors (Supplementary Table 7). Over the first 3 months of lactation, HM lactose concentrations were 2% lower in milk from younger mothers (< 35 vs. ≥ 35 years old, *p* = 0.020) and energy content was 3% lower after delivery by C-section (*p* = 0.034). Also, energy content (*p* = 0.023) and crude protein concentrations (*p* = 0.022) were 4% and 6% higher, respectively, following first childbirth compared to higher order of births (primiparous vs. multiparous). HM macronutrient composition did not differ between maternal ethnicity (Non-Asian vs. Asian), infant sex (Male vs. Female), or infant gestational age (Term/Post-term vs. Preterm).

## 4. Discussion

In our study, HM macronutrients were overall not influenced by the NiPPeR intervention supplement taken preconceptionally and during pregnancy. However, in the subpopulation of control group mothers with overweight/obesity or GDM, fat and energy levels were higher compared to the underweight/normal weight or non-GDM mothers, respectively, over the first 3 months of lactation. Such differences were not observed among the intervention group. Furthermore, among mothers with GDM, the intervention group had lower HM fat and energy levels than the control group. This suggests the impact of GDM status on HM macronutrients was altered by the NiPPeR intervention supplement.

Previous studies have reported that HM macronutrient composition is tightly regulated and is not strongly affected by maternal diet nutritional supplementation (2, 25, 58), with the exception of fatty acids (22). As such, the NiPPeR intervention supplement consumed in the pre-lactation period was not expected to strongly impact HM total fat, energy, lactose, and protein concentrations. Some studies have reported a positive association between maternal BMI and HM fat and energy (23–26), as observed in the present study. The impact of maternal GDM on HM macronutrients is less well understood. Among women with GDM, some have observed higher carbohydrate in colostrum (33), energy in colostrum, transitional, and mature milk (27), while others have reported lower fat and energy content in mature milk (34). Differences between study findings may be due to pre-analytical variations in HM collection protocols and processing. Fat is known to be the most dynamic component of HM but regulatory mechanisms underlying fat synthesis or transport in HM are not well understood. It has been speculated that metabolic dysregulation commonly reported in individuals with GDM, such as hyperglycemia, dyslipidemia, and insulin resistance, may contribute to increased HM fat in these mothers (59, 60). In the current study, using standardised collection methods, GDM status was associated with higher HM fat and energy levels in the control group but not in the intervention group. This suggests that the associations between GDM and HM macronutrients could have been modified by some components in the NiPPeR intervention supplement. Previous studies have observed that supplementation of myo-inositol (61–63), probiotics (64–67), or zinc (68, 69) for 6–8 weeks in women already diagnosed with GDM at 24–28 weeks of gestation, improved glycemic control in these women, as reflected in lower maternal circulating insulin, glucose, triglycerides, total and LDL-cholesterol, and increased insulin sensitivity. In the current study, we speculate that myo-inositol, probiotics, and zinc components in the NiPPeR intervention supplement, postulated to act as insulin sensitizers, modified glucose or lipid metabolism in these mothers, leading to altered HM macronutrient composition. Further studies are required to assess the potential benefits of lower HM fat and energy for infant outcomes, particularly as these relate to growth during infancy and adiposity later in life.

The average HM fat, energy, lactose, and protein concentrations over 12 months observed in the current study are comparable to those reported previously: fat 3.0–4.0 g/100 mL, energy 61–65 kcal/100 mL, lactose 6.6–7.1 g/100 mL, and protein 0.9–1.4 g/100 mL (70–72). We also observed that HM macronutrients displayed various patterns of change over 12 months of lactation. As observed in previous studies, HM lactose remained relatively constant (19, 70, 73), and crude protein decreased (19, 32, 70, 74) until 6 months. Conversely, HM fat initially increased until 3 months, decreased from 3 to 6 months, then increased again from 6 to 12 months of lactation with HM energy content following a similar trajectory. Others have also observed a decrease in HM fat in early lactation followed by an increase in later stages of lactation (73, 75), reporting a positive correlation between HM fat and lactation stage (76). It has been suggested that such changes in HM fat are related to adaptation to changes in infant feeding and energy requirements during development. From about 6 months of age, infants start eating solid foods and breastfeeding becomes complementary. As a result, a reduction in milk volume could be counterbalanced by an increase in fat content to provide sufficient energy for the infant.

Previous studies have investigated the potential influences of maternal ethnicity (17), maternal age (27–30), infant gestational age (21, 27, 30–32), infant sex (26, 35–38), parity (28, 39), and mode of delivery (24, 32, 36, 40) on HM macronutrient composition. However, the results are conflicting, and the underlying mechanisms are not well understood. It has been suggested that anatomical changes of the mammary gland with maternal age (30, 77) and successive pregnancies (28), different hormonal releases associated with mode of delivery (78) and gestational age (79), and different energy demands according to infant sex (35) could contribute to altered HM macronutrient composition. In the current study, we did not observe differences in HM macronutrients according to maternal ethnicity, infant sex, or gestational age. Although we observed a relationship between maternal age and lactose, delivery mode and energy, and parity and energy and crude protein, the magnitude of differences ranged from 2% to 5% in the first 3 months of lactation. While these were statistically significant observations, further research is required to understand the physiological significance of such small absolute changes in HM macronutrient levels in relation to infant outcomes.

## 5. Strengths and limitations

This study has a few strengths to note: (i) HM macronutrient composition was examined from a large cohort of diverse ethnic groups, (ii) standardised HM sample collection, processing, and analytical methods were used, and (iii) the visit windows were tightly controlled, each time point being a distinctive stage of lactation. As longitudinal samples could not be collected from all participants, a repeated measures design was used for statistical analyses. There are some limitations to be acknowledged in the present study. The IADPSG criteria was used for determining GDM status and site-specific diagnostic criteria were not considered. Also, treatment for GDM was an independent decision by the local clinicians and specific for each site. While diet treatment was more common in Singapore, medication treatment with insulin or metformin was more common in New Zealand. Due to the imbalances in sample sizes for treatment types between control and intervention groups, and between sites, any potential impact of GDM treatment modality on HM macronutrients could not be assessed. Finally, infants of this cohort were born generally healthy, none under 28 weeks gestation (extremely preterm) and only 24 infants (7.1%) had low birth weight (< 2,500 g). This precluded investigation of potential influences of extreme infant characteristics on HM macronutrients.

## 6. Conclusion

In this study, we observed that maternal overweight/obesity and GDM were associated with increased HM fat and energy levels among controls but not in the intervention group. This suggests that the intervention supplement during preconception and pregnancy altered the impact of a high maternal BMI and GDM status on HM macronutrient composition. Further studies are required to identify the components in the intervention supplement associated with HM macronutrient composition, characterise the underlying mechanisms, and determine any long-term effects on offspring health.

## Data availability statement

The datasets presented in this article are not readily available because public sharing of the data was not part of the original participant informed consent. Requests to access the datasets should be directed to the corresponding author.

## Ethics statement

The studies involving humans were approved by ethics committees at each study site: Southampton - Health Research Authority National Research Ethics Service Committee South Central Research Ethics Committee (15/SC/0142), Singapore - the National Healthcare Group Domain Specific Review Board (2015/00205), and New Zealand - Northern A Health and Disability Ethics Committee (15/NTA/21).

## Author contributions

SMH: Formal analyses, Investigation, Writing – original draft. JGBD: Formal analyses, Writing – review & editing. MHV: Writing – review & editing. SD: Writing – review & editing. FH: Writing – review & editing. WWP: Writing – review & editing. KMG: Funding acquisition, Writing – review & editing. S-YC: Funding acquisition, Writing – review & editing. SKT: Writing – review & editing, Supervision. WSC: Funding acquisition, Supervision, Writing – review & editing.

## NiPPeR study group

The NiPPeR Study Group authors are: Benjamin B. Albert, b.albert@auckland.ac.nz; Shelia J. Barton, S.J.Barton@soton.ac.uk; Aristea Binia, aristea.binia@rdls.nestle.com; Mary Cavanagh, m.cavanagh@auckland.ac.nz; Hsin Fang Chang, hsin_Fang_Chang@nuhs.edu.sg; Yap Seng Chong, obgcys@nus.edu.sg; Mary F. Chong, mary_chong@nus.edu.sg; Cathryn Conlon, C.Conlon@massey.ac.nz; Cyrus Cooper, cc@mrc.soton.ac.uk; Paula Costello, pc@mrc.soton.ac.uk; Vanessa Cox, vac@mrc.soton.ac.uk; Christine Creagh, christine.creagh@auckland.ac.nz; Marysia Depczynski, m.depczynski@auckland.ac.nz; Sarah El-Heis, se@mrc.soton.ac.uk; Judith Hammond, j.hammond@auckland.ac.nz; Zhang Han, Zhang_Han@sics.a-star.edu.sg; Nicholas C. Harvey, nch@mrc.soton.ac.uk; Mrunalini Jagtap, mrunalini.jagtap1@gmail.com; Timothy Kenealy, t.kenealy@auckland.ac.nz; Heidi Nield, hn@mrc.soton.ac.uk; Justin M. O’Sullivan, justin.osullivan@auckland.ac.nz; Gernalia Satianegara, gernalia_satianegara@sics.a-star.edu.sg; Irma Silva-Zolezzi, Irma.SilvaZolezzi@rdls.nestle.com; Shu E. Soh, shu_e_soh@nuhs.edu.sg; Vicky Tay, Vicky_tay@sics.a-star.edu.sg; Rachael Taylor, rachael.taylor@otago.ac.nz; Elizabeth Tham, elizabeth_tham@nuhs.edu.sg; Philip Titcombe, pt6g13@soton.ac.uk; Clare Wall, c.wall@auckland.ac.nz; Ray Wong, csd3589@yahoo.com; Gladys Woon, gladys_woon@nuhs.edu.sg.
