# Supplementary material for: A nutritional supplement taken during preconception and pregnancy influences human milk macronutrients in women with overweight/obesity and gestational diabetes mellitus
Source: Front Nutr. 2023 Oct 17;10:1282376. doi: 10.3389/fnut.2023.1282376 (PMC10616264; doi:10.3389/fnut.2023.1282376)
Supplement: Supplementary file 1 [file Data_Sheet_1.pdf]

Supplementary Material

Supplementary Table 1. The inclusion, exclusion, and withdrawal criteria for participants in the NiPPeR study (adapted from Godfrey et al. Trials 2017;18:131).

Inclusion criteria	Exclusion criteria	Withdrawal criteria
• Women aged 18-38 years<br>• Living in Southampton, Singapore, or Auckland<br>• In Southampton and Auckland, planning to have future maternity care in Southampton and Auckland, respectively<br>• In Singapore, willing to deliver at the National University Hospital<br>• Planning to conceive within 6 months (although conception up to 12 months after phenotyping deemed acceptable)<br>• In Singapore, being of Chinese, Malay, or Indian ethnicity, or of mixed Chinese/Malay/Indian ethnicity<br>• Ability to provide written informed consent	• Pregnant or currently breastfeeding<br>• Assisted fertility apart from those taking clomiphene or letrozole alone<br>• Pre-existing type 1 or type 2 diabetes (defined as a fasting plasma glucose concentration ≥7.0 mmol/L or post OGTT 2-hour plasma glucose concentration ≥11.1 mmol/L)<br>• Oral or implanted contraception currently or in the last month, or with an intrauterine contraceptive device in situ<br>• Use of metformin or systemic steroids currently or in the last month<br>• Use of anticonvulsant medication currently or in the last month<br>• Treatment for HIV, Hepatitis B or C currently or in the last month<br>• Known serious food allergy	• Participant wishes to withdraw from the study<br>• Participant unwilling or unable to comply with the study protocol (including attendance at study visits, undergoing clinical assessments or biosampling)<br>• An overall uptake level of intervention/control nutritional drink of less than 60% evidenced by sachet counting, each case reviewed by the investigators.<br>• Pregnancy before or at preconception visit 2 (4-6 weeks after randomisation)<br>• Miscarriage (pregnancy loss before 24 weeks of gestation) or ectopic pregnancy. In the event of a first-trimester pregnancy loss, participant could re-join the study with the same randomisation code<br>• Multiple pregnancy (twins or other multiples)<br>• Infant death in the perinatal period (for post-birth secondary outcomes)<br>• Any adverse reaction deemed by the investigators to be causally related to the intervention<br>• Withdrawal at the discretion of the investigators for medical reasons

Supplementary Table 2. Number of participants according to reason for exclusion from the NiPPeR study by site.

Study phase	Reason for exclusion	Control		Intervention	
		Singapore	New Zealand	Singapore	New Zealand
Pregnancy	Miscarriage (≤ 24 weeks gestation)	20	14	18	21
	Twin pregnancy	1	1	1	2
	Ectopic pregnancy	1	2	1	–
	Termination*	2	1	–	–
	Other medical reasons	–	1	–	1
	Lost to follow-up	2	2	3	2
Birth	Lost to follow-up	2	1	2	–
	Infant's medical reasons	–	1	–	1
	Stillbirth	–	–	–	1

* Includes one case of Klinefelter syndrome and other unknown reasons.

Supplementary Table 3. Number of values excluded from analyses for each human milk macronutrient measured in the NiPPeR study.

Macronutrient	Outside the range of the mean ± 5 x SD (n)
Fat	nil
Energy	nil
Lactose	10
Crude protein	1

SD, standard deviation.
In total, 17 samples with at least one '0' measurement (out of 1181 samples or 1.4%) were excluded from analyses due to the likelihood of a sample dilution issue.

Supplementary Table 4. Baseline and perinatal characteristics of 336 participants in Singapore and New Zealand in the NiPPeR study who provided at least one human milk in the first 12 months of lactation.

PARAMETERS	LEVELS	SINGAPORE	NEW ZEALAND	*P*-VALUE
n		158 (47.0%)	178 (53.0%)	
**Adherence (%)**		89.6 (83.3 – 95.3)	90.1 (82.8 – 96.4)	n.s.
**Duration of supplementation (days)**		395 ± 101	403 ± 102	n.s.
**Ethnicity**	Caucasian	–	135 (75.8%)	< 0.001
	Chinese	124 (78.5%)	15 (8.4%)	
	South Asian	12 (7.6%)	8 (4.5%)	
	Malay	20 (12.7%)	–	
	Other	2 (1.3%)	20 (11.2%)	
**Maternal age at delivery (years)**		31.8 ± 2.9	32.4 ± 3.2	n.s.
**Maternal age at delivery group**	< 35 years	134 (84.8%)	141 (79.2%)	n.s.
	≥ 35 years	24 (15.2%)	37 (20.8%)	
**Maternal pre-pregnancy BMI (kg/m^2)**		22.8 ± 4.6	24.7 ± 5.0	< 0.001
**Maternal pre-pregnancy BMI status**	Underweight/normal weight	126 (79.7%)	117 (65.7%)	0.019
	Overweight	19 (12.0%)	37 (20.8%)	
	Obesity	12 (7.6%)	24 (13.5%)	
	Missing	1 (0.6%)	–	
**Highest level of education**	Bachelor's degree or higher	125 (79.1%)	146 (82.0%)	n.s.
	Lesser qualification*	33 (20.9%)	32 (18.0%)	
**Household income quintile**	5 (lowest)	4 (2.5%)	1 (0.6%)	< 0.001
	4	23 (14.6%)	5 (2.8%)	
	3	53 (33.5%)	33 (18.5%)	
	2	48 (30.4%)	67 (37.6%)	
	1 (highest)	24 (15.2%)	62 (34.8%)	
	Missing	6 (3.8%)	10 (5.6%)	
**Smoking during pregnancy**	None	124 (78.5%)	157 (88.2%)	n.s.
	Passive	30 (19.0%)	19 (10.7%)	
	Active	4 (2.5%)	2 (1.1%)	
**Gestational diabetes mellitus**	No GDM	93 (58.9%)	149 (83.7%)	< 0.001
	GDM	55 (34.8%)	28 (15.7%)	
	Excluded	10 (6.3%)	1 (0.6%)	
**Mode of delivery**	Vaginal delivery	123 (77.8%)	120 (67.4%)	0.033
	Caesarean section	35 (22.2%)	58 (32.6%)	
**Infant birth weight (kg)**		3.0 ± 0.44	3.4 ± 0.52	< 0.001
**Infant birth weight status**	Appropriate for gestational age	132 (83.5%)	153 (86.0%)	< 0.001
	Large for gestational age	1 (0.6%)	15 (8.4%)	
	Small for gestational age	25 (15.8%)	10 (5.6%)	
**Infant gestational age (weeks)**		38.8 ± 1.5	39.6 ± 1.5	< 0.001
**Infant gestational age group**	Preterm	14 (8.9%)	11 (6.2%)	n.s.
	Term or post-term	144 (91.1%)	167 (93.8%)	
**Parity**	Primiparous	83 (52.5%)	125 (70.2%)	0.001
	Multiparous	75 (47.5%)	53 (29.8%)	
**Infant sex**	Male	78 (49.4%)	76 (42.7%)	n.s.
	Female	80 (50.6%)	102 (57.3%)	

Data are n (%), mean ± standard deviation (SD), or median (Q1 – Q3). p-values from independent samples *t*-test for continuous variables or Chi-square tests for categorical variables. Adherence to the study protocol was determined by sachet counting. Duration of supplementation calculated by counting the number of days from randomization date to delivery date. Body mass index (BMI) status was defined as per World Health Organisation: underweight/normal weight < 25.0 kg/m^2, overweight 25.0–29.99 kg/m^2, obesity ≥ 30.0 kg/m^2 (World Health Organ Tech Rep Ser 2000;894:i-xii, 1-253). Gestational diabetes mellitus (GDM) was defined by International Association of Diabetes and Pregnancy Study Groups criteria (Diabetes Care 2010;33:676-82). Birth weight categories were determined using the Royal College of Paediatrics and Child Health 2009 U.K.-World Health Organization growth charts (Ann Hum Biol 2011;38(1):7-11). Gestational age was determined using a pre-specified algorithm as previously described (Thorax 2010;65:1099–106) with preterm defined as birth < 37 weeks of gestation, and term or post-term as birth at ≥ 37 weeks of gestation. *Including incomplete and complete high school qualifications, and other tertiary level qualifications below bachelors (e.g. diploma or certificate). n.s., not statistically significant at p<0.05.

Supplementary Table 5. The average macronutrient concentrations in human milk over the first 3 months of lactation in intervention and control groups in the NiPPeR Study.

	Intervention	Control	aMD	p-value
**Overall**				
Fat (g/100mL)	3.81 (3.65, 3.98)	3.71 (3.55, 3.87)	1.03 (0.97, 1.09)	0.367
Energy (kcal/100mL)	70.3 (68.9, 71.8)	69.8 (68.4, 71.3)	1.01 (0.98, 1.04)	0.609
Lactose (g/100mL)	7.07 (7.01, 7.12)	7.05 (7.00, 7.11)	1.00 (0.99, 1.01)	0.745
Crude protein (g/100mL)	1.29 (1.25, 1.33)	1.32 (1.28, 1.36)	0.97 (0.93, 1.02)	0.243
**Singapore**				
Fat (g/100mL)	3.88 (3.68, 4.09)	3.67 (3.48, 3.87)	1.06 (0.98, 1.14)	0.153
Energy (kcal/100mL)	70.7 (68.9, 72.6)	69.5 (67.7, 71.4)	1.02 (0.98, 1.06)	0.354
Lactose (g/100mL)	7.02 (6.94, 7.10)	7.03 (6.95, 7.11)	1.00 (0.98, 1.02)	0.890
Crude protein (g/100mL)	1.28 (1.23, 1.33)	1.33 (1.28, 1.38)	0.96 (0.91, 1.02)	0.162
**New Zealand**				
Fat (g/100mL)	3.75 (3.49, 4.04)	3.82 (3.56, 4.09)	0.98 (0.89, 1.09)	0.751
Energy (kcal/100mL)	70.3 (67.9, 72.8)	70.7 (68.5, 73.0)	0.99 (0.95, 1.04)	0.815
Lactose (g/100mL)	7.12 (7.04, 7.21)	7.07 (6.99, 7.14)	1.01 (0.99, 1.02)	0.322
Crude protein (g/100mL)	1.29 (1.22, 1.36)	1.30 (1.24, 1.37)	0.99 (0.92, 1.07)	0.826

Data are least-squares means (i.e., adjusted mean) for each group or the adjusted mean difference (aMD) and respective 95% confidence intervals derived from repeated measures analyses, adjusted for randomisation group, visit, their interaction term (group*visit), study site (for overall only), maternal pre-pregnancy BMI, gestational age at birth, and adherence. All data have been log-transformed to approximate a normal distribution and then back-transformed; thus, the aMD represents a proportional difference between the groups (i.e., intervention vs control).

Supplementary Table 6. Overall macronutrient concentrations in human milk of participants in New Zealand in the NiPPeR study, during the first 12 months of lactation.

Macronutrient	1 week	3 weeks	6 weeks	3 months	6 months	9 months	12 months
Fat (g/100mL)	3.71 ± 1.39	3.91 ± 1.00	4.03 ± 1.22	4.07 ± 1.33	3.79 ± 1.73	4.56 ± 1.61	4.71 ± 1.93
Energy (kcal/100mL)	70.6 ± 12.1	71.0 ± 9.5	71.0 ± 11.1	70.1 ± 12.2	67.2 ± 15.5	73.8 ± 14.6	75.1 ± 17.3
Lactose (g/100mL)	7.15 ± 0.43	7.18 ± 0.35	7.11 ± 0.49	7.01 ± 0.40	7.09 ± 0.37	6.89 ± 0.47	6.89 ± 0.40
Crude protein (g/100mL)	1.75 ± 0.23	1.45 ± 0.26	1.20 ± 0.25	1.02 ± 0.34	0.90 ± 0.29	0.92 ± 0.29	0.95 ± 0.25

Data are the unadjusted means ± standard deviations.

Supplementary Table 7. The average macronutrient concentrations in human milk (HM) over the first 3 months of lactation in various maternal and infant sub-groups.

**Maternal ethnicity**	**Asian**	**Non-Asian**	**aMD**	**p-value**
Fat (g/100mL)	3.80 (3.57, 4.04)	3.70 (3.40, 4.02)	1.03 (0.90, 1.17)	0.683
Energy (kcal/100mL)	70.5 (68.5, 72.6)	69.5 (66.8, 72.2)	1.01 (0.95, 1.08)	0.645
Lactose (g/100mL)	7.08 (7.00, 7.16)	7.03 (6.92, 7.14)	1.01 (0.98, 1.03)	0.073
Crude protein (g/100mL)	1.32 (1.27, 1.38)	1.27 (1.20, 1.35)	1.04 (0.95, 1.14)	0.419
**Maternal age**	**≥ 35 years**	**< 35 years**	**aMD**	**p-value**
Fat (g/100mL)	3.62 (3.37, 3.88)	3.79 (3.66, 3.92)	0.95 (0.88, 1.03)	0.231
Energy (kcal/100mL)	68.4 (66.2, 70.7)	70.5 (69.3, 71.7)	0.97 (0.93, 1.01)	0.112
Lactose (g/100mL)	6.96 (6.87, 7.05)	7.08 (7.04, 7.13)	0.98 (0.97, 1.00)	**0.020**
Crude protein (g/100mL)	1.29 (1.23, 1.36)	1.30 (1.27, 1.34)	0.99 (0.94, 1.05)	0.812
**Delivery mode**	**C-section**	**Vaginal**	**aMD**	**p-value**
Fat (g/100mL)	3.95 (3.39, 3.81)	3.82 (3.69, 3.96)	0.94 (0.88, 1.01)	0.075
Energy (kcal/100mL)	68.3 (66.4, 70.2)	70.8 (69.6, 72.0)	0.97 (0.93, 1.00)	**0.034**
Lactose (g/100mL)	7.01 (6.94, 7.09)	7.08 (7.03, 7.12)	0.99 (0.98, 1.00)	0.182
Crude protein (g/100mL)	1.30 (1.24, 1.35)	1.30 (1.27, 1.34)	1.00 (0.95, 1.05)	0.883
**Parity**	**Multiparous**	**Primiparous**	**aMD**	**p-value**
Fat (g/100mL)	3.61 (3.43, 3.80)	3.84 (3.70, 3.99)	0.94 (0.88, 1.00)	0.056
Energy (kcal/100mL)	68.5 (66.9, 70.2)	71.0 (69.7, 72.2)	0.97 (0.94, 1.00)	**0.023**
Lactose (g/100mL)	7.04 (6.97, 7.11)	7.07 (7.02, 7.12)	1.00 (0.98, 1.01)	0.465
Crude protein (g/100mL)	1.26 (1.21, 1.31)	1.33 (1.29, 1.36)	0.95 (0.91, 0.99)	**0.022**
**Infant sex**	**Female**	**Male**	**aMD**	**p-value**
Fat (g/100mL)	3.71 (3.56, 3.86)	3.82 (3.65, 3.99)	0.97 (0.92, 1.03)	0.363
Energy (kcal/100mL)	69.7 (68.3, 71.0)	70.6 (69.1, 72.2)	0.99 (0.96, 1.02)	0.357
Lactose (g/100mL)	7.05 (7.00, 7.10)	7.07 (7.01, 7.13)	1.00 (0.99, 1.01)	0.651
Crude protein (g/100mL)	1.30 (1.26, 1.34)	1.31 (1.27, 1.35)	0.99 (0.95, 1.04)	0.734
**Gestational age**	**Preterm**	**Term**	**aMD**	**p-value**
Fat (g/100mL)	4.01 (3.61, 4.44)	3.73 (3.61, 3.85)	1.07 (0.96, 1.20)	0.191
Energy (kcal/100mL)	71.6 (68.1, 75.2)	69.9 (68.9, 71.0)	1.02 (0.97, 1.08)	0.372
Lactose (g/100mL)	6.97 (6.84, 7.11)	7.07 (7.03, 7.11)	0.99 (0.97, 1.01)	0.185
Crude protein (g/100mL)	1.35 (1.25, 1.45)	1.30 (1.27, 1.33)	1.04 (0.96, 1.12)	0.333

Data are least-squares means (i.e., adjusted mean) for each group or the adjusted mean difference (aMD) and respective 95% confidence intervals derived from repeated measures analyses, adjusted for randomisation group, visit, their interaction term (group*visit), maternal pre-pregnancy BMI, gestational age at birth, and adherence. All data have been log-transformed to approximate a normal distribution and then back-transformed; thus, the aMD represents a proportional difference between the groups. Bold font indicates statistically significant p-value <0.05.
